# A sciatic nerve gap-injury model in the rabbit

**DOI:** 10.1007/s10856-022-06642-x

**Published:** 2022-01-21

**Authors:** Antonio Merolli, Michelle Li, Gregory Voronin, Lauren Bright

**Affiliations:** 1 Department of Physics and Astronomy, Rutgers—The State University of New Jersey, New Brunswick, NJ USA; 2 New Jersey Center for Biomaterials, Rutgers—The State University of New Jersey, New Brunswick, NJ USA; 3 In Vivo Research Services, Rutgers—The State University of New Jersey, New Brunswick, NJ USA; 4 Comparative Medicine Resources, Rutgers—The State University of New Jersey, New Brunswick, NJ USA

## Abstract

There has been an increased number of studies of nerve transection injuries with the sciatic nerve gap-injury model in the rabbit in the past 2 years. We wanted to define in greater detail what is needed to test artificial nerve guides in a sciatic nerve gap-injury model in the rabbit. We hope that this will help investigators to fully exploit the robust translational potential of the rabbit sciatic nerve gap-injury model in its capacity to test devices whose diameter and length are in the range of those commonly applied in hand and wrist surgery (diameter ranging between 2 and 4 mm; length up to 30 mm). We suggest that the rabbit model should replace the less translational rat model in nerve regeneration research. The rabbit sciatic model, however, requires an effective strategy to prevent and control self-mutilation of the foot in the postoperative period, and to prevent pressure ulcers.

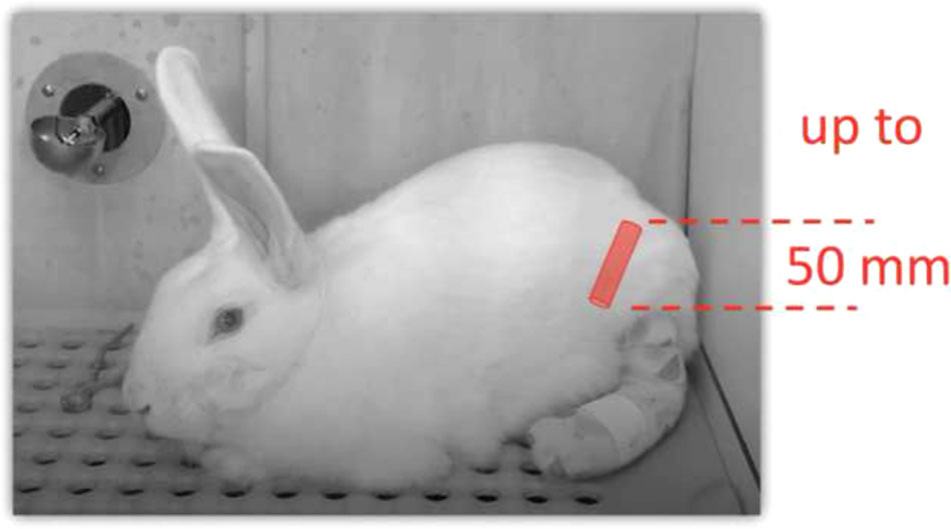

## Introduction

Peripheral nerve regeneration after a nerve-gap injury may be assisted by artificial devices called nerve guides (or “nerve-conduits” or “nerve-cuffs”) [1]. They are implantable devices that bridge the gap and provide a closed space for the protection and guidance of regenerating nerve stumps. Cylindrical nerve guides are routinely used in clinical practice for the treatment of individual nerve gap lesions. They are a reliable alternative to nerve autografting, which is currently regarded as the best clinical option despite having the intrinsic disadvantage of sacrificing a healthy nerve [2]. An overview of the clinical outcome of injuries treated by artificial nerve guides showed that the guides perform just as well as autografts in gaps shorter than 20 mm, bearing the significant advantage of avoiding donor site morbidity [2–5].

The sciatic nerve gap-injury model in the rat is the most used animal model in this research field [6, 7]. It is very affordable in comparison with higher species, so it allows more animals to be tested to achieve a better statistical significance of results. There are several limitations, however, with the use of the rat sciatic model [8, 9]. The small volume, length, and diameter of the rat sciatic nerve restricts the dimensions of the devices that can be tested; this, in turn, reduces the model translational potential. Even though the majority of artificial nerve guides are used in patients suffering from injuries in the small-diameter nerves of the hand and wrist [2], these small nerves are still often larger than the rat sciatic nerve. So far, research on artificial nerve guides has been mostly aimed at obtaining guides that will be more effective in gap lesions longer than 20 mm, but the study of guides longer than 20 mm is impossible in the rat sciatic model, because there is simply not enough space in the rat sciatic compartment. The use of higher species with greater translational capability, like the pig, the sheep, or the nonhuman primate (for example: the macaque), is significantly more complex and expensive though. For this reason, these models are mostly used after initial studies have been performed in the rat. Compared with the rat, there have been a relatively limited number of publications using the rabbit as the species to test nerve injury models. Some authors have addressed the “crush-injury” model [10–19] and not the transection model. Some authors differentiated between the tibial [20–22] and the peroneal [23–27] branches of the sciatic nerve as the site of injury for their model. Several authors used the rabbit to develop nerve injury models of other nerves, like the facial [28] or the median nerve [29, 30]. The use of a rabbit model to test artificial nerve guides has been specifically proposed since the 1990s [31, 32]. In the past 2 years there has been an increase in the number of studies of transection injuries with this model [20, 25, 33–37]. Most of the authors have just briefly described their technique, reporting a kind of scaled-up replica of the method used in the rat (for a review see: [6, 7]). While papers have been published where the rabbit model was used to study a direct repair [38–41] or a 10-mm gap [23, 42–45], the need for a model to test guides as long as 50 mm was a common rationale in the use of the rabbit model. Gaps have been studied as long as 15-mm [24, 46, 47], 20-mm [35, 48, 49], 25-mm [20, 50], 30-mm [25, 26], and 40-mm gap [21, 27, 36]. A recent paper [51], however, has warned about how the rabbit sciatic model requires an effective strategy to prevent and control self-mutilation of the foot in the postoperative period, and to prevent pressure ulcers (which are often underreported complications [50]). It is necessary to address these problems to fully exploit the robust translational potential of the rabbit sciatic nerve gap-injury model in its capacity to test devices whose diameter and length are in the range of those commonly applied in hand and wrist surgery (diameter ranging between 2 and 4 mm; length not longer than 30 mm). Another relevant advantage is that (for the above mentioned reasons) the rabbit model can provide both a first stage testing model for artificial nerve guides but, at the same time, an adequate preclinical model.

We wanted to define in greater detail what is needed to test artificial nerve guides in a sciatic nerve gap-injury model in the rabbit, providing details as specific as the anchoring technique for the stump or the way to cut the nerve, for example. We hope that this model will replace the less translational rat model in the near future. We focused not only on surgery, but we refined and optimized a comprehensive approach which included: postoperative follow-up, prevention and control of self-mutilation and pressure ulcers, implant-retrieval technique and histological analysis. This comprehensive approach was tested in a study on sixteen rabbits, in a 16-week longitudinal study. Here, we will report on the detailed description of the different steps of our approach while providing a real applicative example. The study was centered on a nerve-guide assisted regeneration strategy in guides longer than 20 mm; here we will briefly report the main finding but the detailed specific results will be the subject of a different paper.

## Materials and methods

### Animals

The study population was comprised of 16 male New Zealand White rabbits (Oryctolagus cuniculus; age: 3 m, 8d); all rabbits were obtained from Charles River Laboratories (Attica, MI, USA). This group of rabbits were adult and not in the growth phase, and not expected to gain weight postoperatively in the maximum of 16 weeks period. There were no significant changes in body weight from the day of operation to the day of termination: body weight at the day of operation was 3.325 ± 0.2 kg, and at the day of termination was 3.367 ± 0.3 kg. Animals were single-housed in an AAALAC-accredited facility at 70 °F (21.1 °C), 30–70% humidity, 10–15 air changes hourly, and a 12:12-h light:dark cycle (7am-7pm). Rabbits were fed a standard, nutritionally-complete rabbit diet (5326, LabDiet, St. Louis, MO, USA), and were provided *ad libitum* timothy hay (Oxbow Animal Health, Omaha, NE, USA) and hyperchlorinated water. Health reports from vendor sentinel animals were pathogen-free for the following recognized pathogens: *Pasteurella multocida, Bordetella bronchiseptica, Salmonella* spp*., Helicobacter* sp., *Lawsonia* sp., *Treponema cuniculi, cilia-associated respiratory bacillis, Clostridium piliforme, Encephalitozoon cuniculi, Psoroptes cuniculi, Cheyletiella parasitivorax, Listrophorus gibbus, Passalurus ambiguous, Eimeria* spp., reovirus, lymphocytic choriomeningitis virus, parainfluenza virus 1 and 2, rotavirus, rabbit hemorrhagic disease virus, endoparasites, and ectoparasites. This project was approved by the Institutional Animal Care and Use Committee at Rutgers, The State University of New Jersey, and animals were treated in accordance with the *Guide for the Care and Use of Laboratory Animals* (10.1258/la.2012.150312), and in compliance with USDA regulations. Rabbits were allowed 2 weeks to acclimate to the housing facility prior to initiation of experimental work. All rabbits were deemed healthy by clinical examinations by veterinarians prior to anesthetic induction.

### Analgesia and anesthesia

Analgesia was based on buprenorphine (0.03 mg/kg, SC, every 8–12 h) or buprenorphine SR (0.12 mg/kg, SC, every 48–72 h) administered immediately before surgery. Animals also received buprenorphine (0.03 mg/kg, SC, every 8–12 h) or buprenorphine SR (0.12 mg/kg, SC, every 48–72 h), for postoperative analgesia for up to 3 days. For anesthesia, rabbits were sedated with ketamine (5–10 mg/kg SC) + xylazine (1 mg/kg SC). After sedation, sterile ophthalmic ointment was placed on the eyes to protect the cornea during anesthesia, an intravenous catheter was placed in the marginal ear vein, and glycopyrrolate (0.05 mg/kg SC) was given to reduce airway secretions for intubation. Rabbits were then induced with propofol (21 mg/kg IV) and intubated with a size 3.0 endotracheal tube, after which isoflurane gas anesthesia (2–4% in 100% O2) was initiated to maintain anesthesia. Local anesthetic (bupivacaine 0.25%, not exceeding 2 mg/kg total per animal) was administered along the predicted incision line. Animals received an injection of Baytril (enrofloxacin) antibiotic at 5 mg/kg (SC/PO).

Adequate depth of anesthesia was verified by evaluation for lack of the ear pinch reflex and loss of jaw tone before placing a surgical drape over the animal. During anesthesia, the color of the mucous membranes (eye, lips, tongue), respiratory rate, heart rate and EKG, end tidal CO2, SpO2, and rectal temperature was monitored once every 5 min. Yohimbine (1–2 mg/kg IV) was administered immediately after closure, at veterinary staff discretion, to encourage a speedy recovery from Xylazine. Initially, rabbits were given acetaminophen (96 mg PO) twice daily for a week following surgery, for continued pain relief, but as the project progressed, this was discontinued as it appeared to be a stressor to the animals without any added benefit.

### Surgical approach

**Fig. 1:**
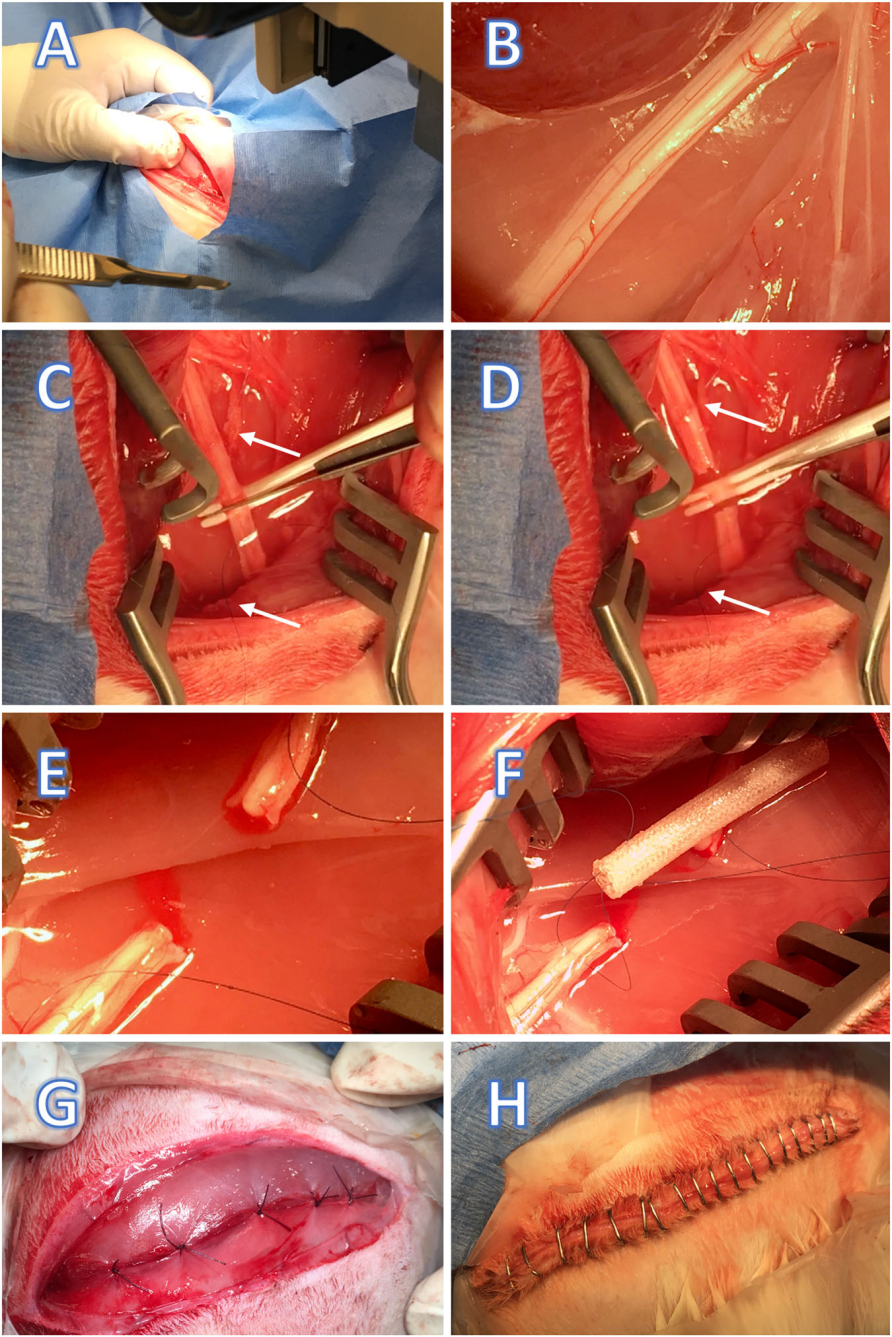
**A** palpatory identification of the reference points; (**B**) visual identification of the sciatic nerve; (**C**–**E**) after a proximal and a distal trans-epineural anchoring was made by a double-arm 7-0 nylon monofilament (arrows), a sharp cut was produced with the assistance of a 3D printed tool; (**F**) a polymeric artificial nerve-guide was implanted; (**G**) a resorbable 5-0 suture was used for the fascia; (**H**) a skin closure with metallic staples completed the procedure

All surgeries were perfomed by a the same individual and with the same support staff. In regard to the 12:12 h light:dark cycle (7 a.m.–7 p.m.), all surgeries where completed by 12 pm. The surgical site (lateral thigh) was shaved (tricotomized) and local anesthetic was injected SC. The shaved area was then scrubbed with three washes of alternating applications of chlorhexidine scrub followed by 70% isopropyl alcohol. Full sterile surgical technique was used. With the animal in right lateral recumbency, surgical landmarks were established by palpation of anatomic reference points (lateral condyle of the knee; greater trochanter, Fig. 1A). After sterile draping of the area, a linear longitudinal postero-lateral incision was made by a #15 blade. Then incision of the fascia and smooth dissection between the anterior and posterior muscular compartments followed. Deep access and visual identification of the sciatic nerve completed the approach (Fig. 1B). A proximal and distal trans-epineural anchoring by a double-arm 7–0 nylon monofilament suture was performed before producing the experimental lesion. A sharp transverse section of nerve was performed with the help of a disposable 3D printed tool (called TASC, see below) and a #11 blade (Fig. 1C–E). Then followed the proximal and distal anchoring of a braided Tyrosine-derived polymeric artificial nerve-guide [52] of 3 mm inner diameter (Fig. 1F). There were eight implants of 24 mm in length and 8 of 44 mm in length in the experimental design. The proximal and distal nerve stumps were then gently inserted inside the guide by pulling the 7-0 suture transepineurial anchoring. A double knot secured the stump in place. Saline irrigation accompanied all steps. A braided resorbable 5-0 suture of the fascia (Fig. 1G) and a skin closure with metallic staples completed the procedure (Fig. 1H). Animals were kept on a heated pad for the entire procedure until they were fully recovered from anesthesia.

### Instrument for the tool assisted sharp cut (TASC)

**Fig. 2:**
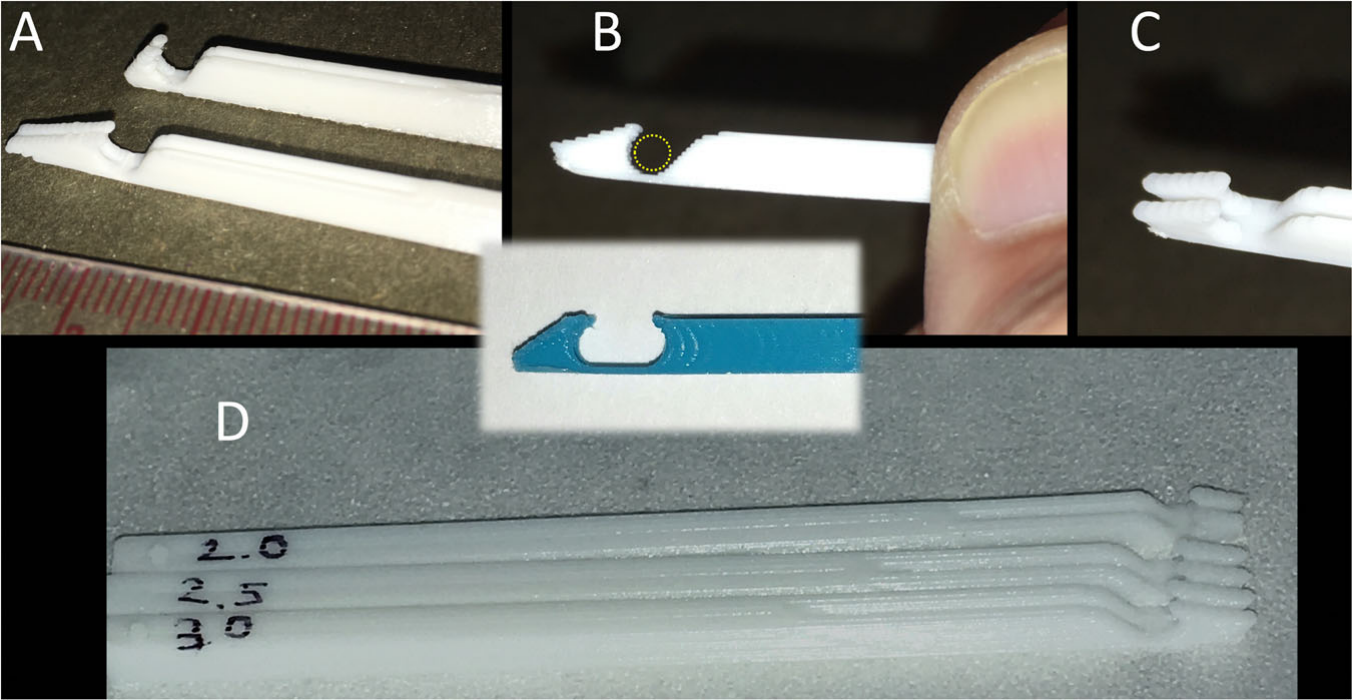
**A** several profiles were tested to optimize the design of an instrument for the Tool Assisted Sharp Cut (TASC); (**B**) the probe accommodates the nerve transverse section (dotted circle) into an ellipsoid compartment (inset) which is open on the top for about 1/3 of its circumference; (**C**) a slit is present with dimensions apt to position and guide the surgical blade; (**D**) the instrument was 3D printed in several sizes for different nerve diameters

We designed a 3D printed disposable cutting probe to consistently achieve uniform transverse sharp cuts. The probe with a double-profiled tip (Fig. 2A) was designed to accommodate the nerve transverse section into an ellipsoid compartment which is open on the top for a length equaling the diameter of its circular compartment (Fig. 2B and inset). A slit is present with dimensions apt to position and guide the surgical blade (Fig. 2C). The instrument was 3D printed with poly-Lactic Acid, and in several sizes for application in different nerves (Fig. 2D). Diameter used for the rabbit sciatic nerve was 3.0 mm. It was sterilized by Ethylene Oxide.

### Postoperative follow-up

Baytril (5 mg/kg, SC/PO) was administered for up to 3 days postoperatively, if indicated at the advice of the Veterinary staff. At least one of the following options was used for postoperative pain relief: Buprenorphine (0.03 mg/kg, SC, every 8–12 h), Buprenorphine SR (0.12 mg/kg, SC, every 48–72 h), Meloxicam (0.3–0.6 mg/kg, SC, every 24 h), Meloxicam SR (0.6 mg/kg, SC, every 48–72 h), and/or Carprofen (4 mg/kg SC every 24 h). These drugs were administered following implantation and were continued for at least 3 days. Metallic staples were removed after healing, which occurred 10–14 days post-operatively.

### Long-term follow-up

Animals were housed individually as a precaution to avoid trauma of and interference with the surgical site by other animals in the same cage. Animals were monitored at least two times a day for the first 3 days for any signs of pain or distress (within 2 h of lights-on and 2 h of lights-off, relative to the light:dark cycle). Afterwards, they were monitored at least once a day until staple removal.

### Prevention and control of self-mutilation behavior

**Fig. 3:**
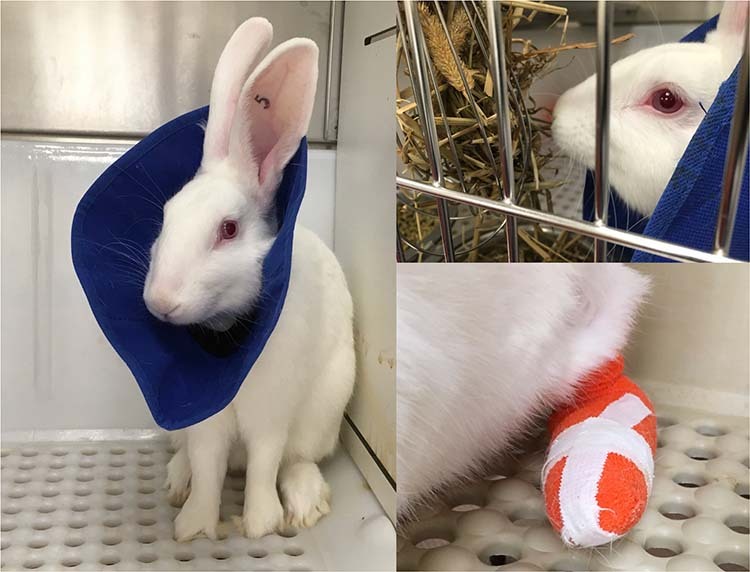
A combined approach to prevent self-mutilation and/or treat its consequences was based on: (1) a training of the rabbits to continue their daily activities while wearing a soft Elizabethan (e-collar); (2) bandaging the surgical limb with tape stirrups

We implemented a combined approach to prevent self-mutilation and/or treat its consequences. Prevention was based on: (1) acclimating rabbits to a soft Elizabethan (e-collar) 3 days prior to surgery for 4–6 h each day. This was aimed at having the rabbits adjusted to wearing the e-collar in preparation for its use during the immediate 24–72 h post-surgery. (2) Bandaging the surgical limb with tape stirrups, Telfa^TM^ pads (Medtronic, Minneapolis, MN, USA), roll gauze, and VetWrap^TM^ (3M^TM^, USA) (Fig. 3). Collars were utilized following surgery for 3 days and then removed if there were no signs of self-mutilation. Prophylactilly, the collar and bandage were placed again in cases where the rabbits exhibited signs of self-trauma, or showed increased interaction with the foot.

### Euthanasia and implant retrieval

**Fig. 4:**
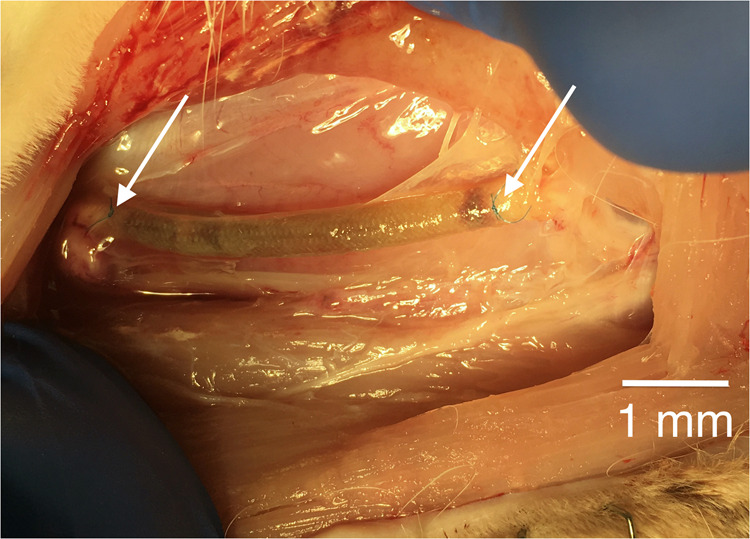
The cylindrical guide (conduit) exposed at retrieval; not-resorbable suture are visible (arrows)

**Fig. 5:**
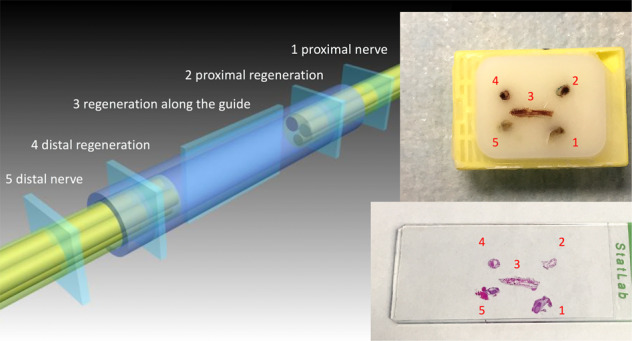
Sampling procedure defined five regions of interest: 1-the transverse section of the proximal stump about 1 mm proximal to the lesion; 2-the transverse section of the regenerated tissue about 1 mm distal to the proximal stump; 3-the longitudinal section of the artificial nerve-guide; 4-the transverse section of the regenerated tissue about 1 mm proximal to the distal stump; 5-the transverse section of the distal stump about 1 mm distal to the lesion. Sampled regions were embedded into paraffin (upper inset) and positioned according to a fixed scheme that helps in standardizing the histological reading (lower inset)

The animal was placed in a rabbit restraint box/device, sling or manually restrained, and then sedated with ketamine (35 mg/kg IM) + xylazine (5 mg/kg IM). Once the animal was nonresponsive to an aversive stimulus (toe pinch), alcohol was applied to the skin of the ear and the ear was held to allow for venous stasis and distention. The vein was punctured with a needle/syringe or butterfly cannula, 20–23 gauge. Once blood was observed in the syringe/cannula, the hold was released and pentobarbital (150–200 mg/kg) was administered. The animal was confirmed deceased by cardiac auscultation and corneal reflex, then retrieval of the implant followed immediately. By the same surgical access used for implantation, the guide was exposed (Fig. 4) and retrieved en bloc together with about 5 mm of the proximal and distal nerve stumps. Sampling procedure defined five regions of interest on the nerve (as we described a similar approach in the rat [53]). These were: 1-the transverse section of the proximal stump about 1 mm proximal to the lesion; 2-the transverse section of the regenerated tissue about 1 mm distal to the proximal stump; 3-the longitudinal section of the artificial nerve-guide; 4-the transverse section of the regenerated tissue about 1 mm proximal to the distal stump; 5-the transverse section of the distal stump about 1 mm distal to the lesion (Fig. 5).

### Histological methods

The sampled regions of interest were embedded into paraffin and positioned according to a fixed scheme that helps in standardizing the histological reading (Fig. 5). This allowed for a systematic and comparative reading of the results among each region of the nerve and among different nerve samples.

**Fig. 6:**
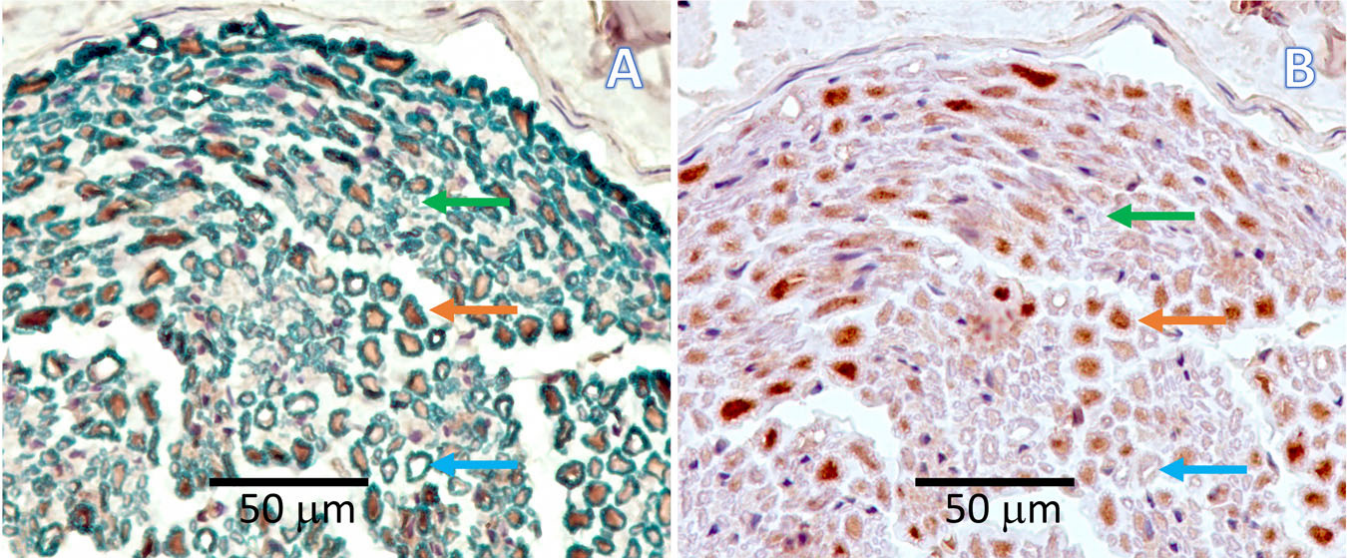
**A** An on-slide procedure for the sequential double nonfluorescent immunostaining on paraffin sections (RNS: reciprocal nerve staining) showed a good contrast between choline acetyltransferase reaction product of motor fibers (light brown arrow) and myelin basic protein reaction product (green arrow); in this way, myelinated sensory choline acetyltransferase negative fibers (light blue arrow) are better recognizable than in the (**B**) traditional choline acetyltransferase single staining procedure

Standard Hematoxylin & Eosin and Masson Trichrome staining protocols were used to visualize cells and collagen fibers respectively. To analyze myelinated fibers, we developed an on-slide procedure for the sequential double nonfluorescent immunostaining on paraffin embedded sections (RNS: reciprocal nerve staining) [54]. In this procedure, incubation in sheep polyclonal choline acetyltransferase antibody (Abcam 18736) at dilution of 1:150 was followed by incubation in mouse monoclonal anti-myelin basic protein antibody (Abcam 62631) at a dilution of 1:5000. Counterstaining was performed with hematoxylin QS (Vector Labs H-3404). This combination showed a good contrast between the light brown of the choline acetyltransferase reaction product and the green of myelin basic protein reaction product (cell nuclei are stained blue) (Fig. 6). Glial fibrillary acidic protein (GFAP) was used to visualize not-myelinating Schwann cell. Sections were incubated for 1 h with mouse monoclonal anti-GFAP protein antibody (Abcam 212401) at a dilution of 1:2000. Then they were incubated with secondary antibody for 30 min with ImmPRESS VR Horse anti-mouse IgG HRP Polymer Detection Kit (Vector Labs MP-6402). Immunostaining procedure was completed by reacting the sections for approximately 5 min in ImmPACT VIP Substrate, Peroxidase (HRP) chromogen (Vector Labs SK-4605). Counterstaining was performed with hematoxylin QS (Vector Labs H-3404).

Slides were scanned by an Aperio CS2 slide scanner and processed with Aperio ImageScope software (Leica Biosystems). Post-processing was accomplished using NIH-Fiji software.

## Results

### Duration of surgery

The mean operative time (from incision to closure) was 39 ± 9 min. This time included about 8 min of video/photography recording.

### Efficacy of the “Tool assisted sharp cut” (TASC)

The smooth tip allowed the positioning of the instrument in the area designated for the cut. Availability of probes of different sizes allowed the precise matching of the diameter of the nerve with its circular compartment. The slit provided a constraint for the surgical blade to cross the nerve at right angle with the elongation of the nerve fibers. The instrument allowed a fast and accurate sharp cut of the nerve. The manufacturing technique (3D printing) provided an easy way to produce several different diameters available for surgery. The disposable character of the instrument eliminated the need for any cleaning of the slit from biological materials (as it would be required for the re-use of more traditional non-disposable tools). By using the TASC, the nerve remains in position to be transversely cut and the blade cannot displace it. Video-recordings of the cutting procedure showed that the nerve stumps retract in <0.1 s.

### Postoperative response

Animals showed no signs of distress while recovering after surgery. Active, purposeful locomotion was regained rapidly after termination of anesthetic delivery. Drinking and eating resumed at the same time.

### Prevention and control of the self-mutilation

**Fig. 7:**
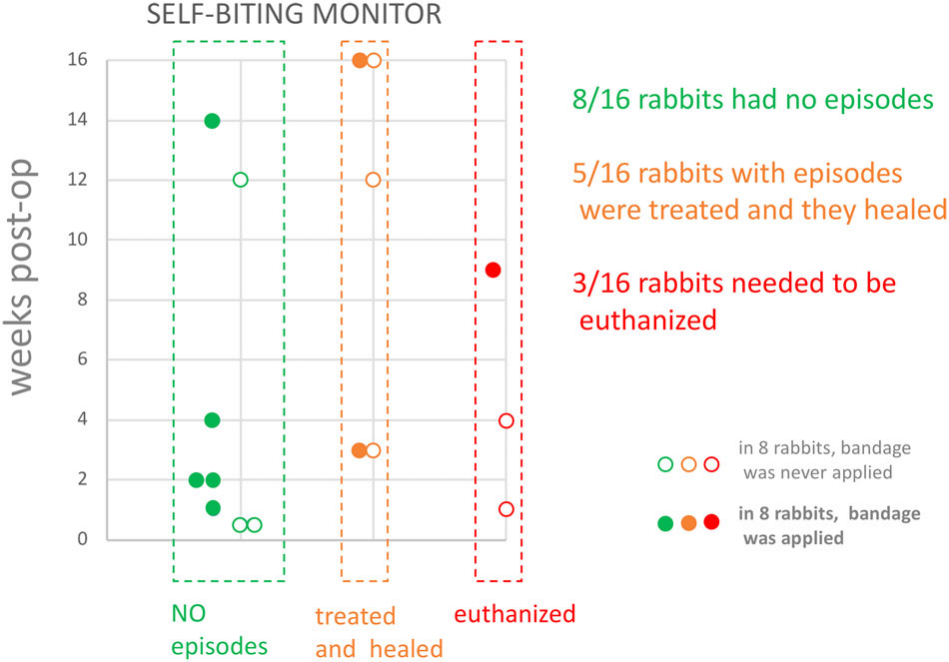
Episodes of self-mutilation showed that half of the rabbits did not exhibit any self-mutilation behavior at all. Five self-mutilation episodes were treated with medication and were followed by healing. Three self-mutilation episodes led to the rabbit’s euthanasia. Self-mutilation episodes were quite evenly distributed along the 16 weeks. Prophylactic bandaging of the surgical limb was utilized in half of the rabbits but an acute episode was observed even with the presence of the bandage and required euthanasia

Monitoring the episodes of self-mutilation (Fig. 7) showed that half of the rabbits (8 out of 16) did not exhibit any self-mutilation behavior at all. In the remaining half, episodes of self-mutilation were present in a variable degree of severity, from simple chewing on one or more toe nails to self-removal of a distal phalanx with exposure of the inter-digital joint. Five of eight self-mutilation cases resolved with treatment. Three of eight self-mutilation cases led to the rabbit’s euthanasia. The reason for self-mutilation behaviors was not entirely clear, but was not associated with nerve-guide length as there were 4 cases in the group with 24 mm guides and 4 in the group with 44 mm guides. Self-mutilation episodes were also quite evenly distributed along the 16 weeks duration of the experiment, showing that they can occur anytime. Prophylactic bandaging of the surgical limb was utilized in half of the rabbits (8 out of 16). Throughout the study we did observe some bandages that were chewed by the rabbits, but they had stopped before they got access to the foot. It is notable that an acute self-mutilation episode was observed even in the presence of the bandage (and required euthanasia).

### Control of pododermatitis and incisional dehiscence

In addition to self-mutilation episodes, we also observed pododermatitis of the surgical limb severe enough to require treatment in 3 out of 16 rabbits. This treatment consisted of cleaning the affected area, applying silver sulfadiazine ointment, bandaging the affected limb, ±NSAID administration. This pododermatitis is thought to be secondary to a shift in the limb placement following the nerve transection. This change over time resulted in ulcerative pododermatitis on the plantar surface of the heel on the affected limbs. Rabbits wearing a bandage had decreased incidences of pododermatitis, likely due to the protective layer of the bandage. Another adverse event observed was incisional dehiscence, secondary to rabbits chewing the incision. This was treated by cleaning, closing the incision if applicable, and placing an Elizabethan collar until sutures were removed at 14 days.

### Sampling procedure

Qualitatively, the five regions of interest provided replicate documentation of the regeneration of the nerve and the concurrent proximal and distal (Wallerian) degeneration of the stumps. There was often an overlap of histological features between the transverse Section 1 mm proximal (region 1) or distal (region 5) to the lesion and the transverse section of the regenerate (region 2 and 4). At the same time, the regions 2 and 4 have overlapping features with the longitudinal section of the regenerate (region 3). Reciprocal Nerve Staining was effective in differentiating myelinated motor fibers from myelinated sensory fibers, as observed as reported elsewhere [54].

### Nerve regeneration

**Fig. 8:**
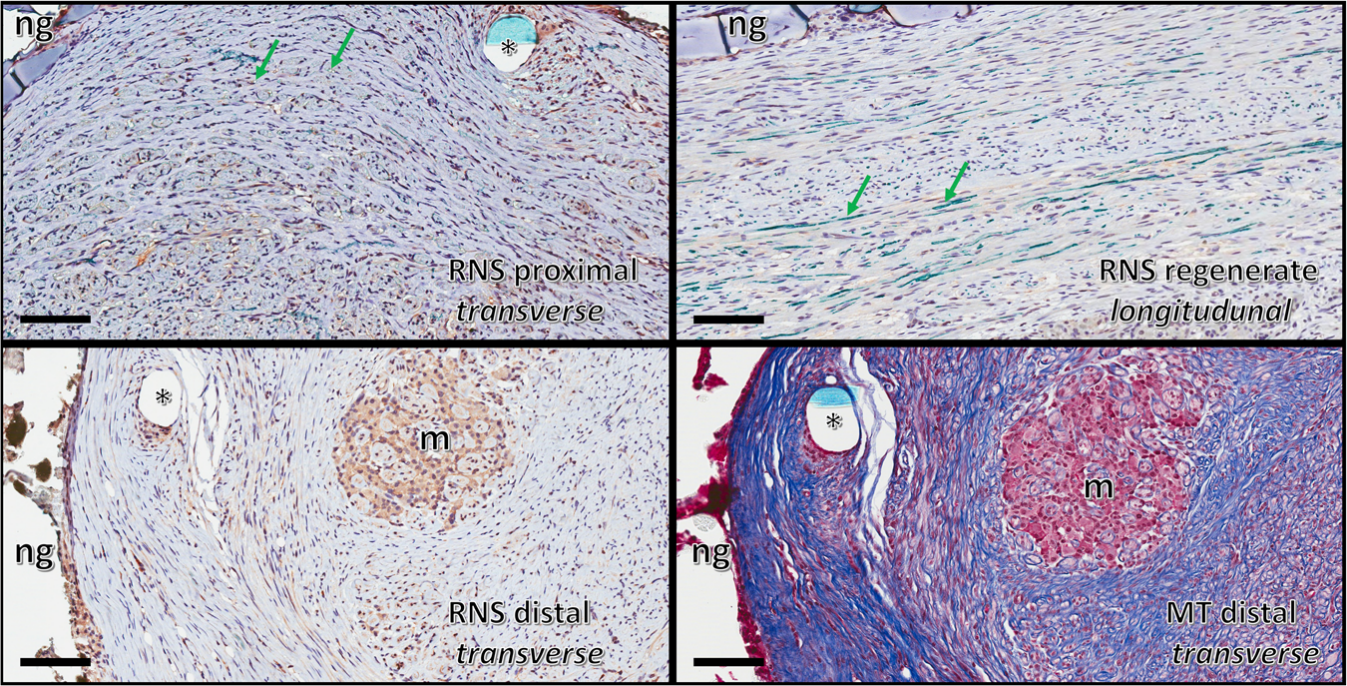
The RNS staining of the region 1, 3, and 5 (proximal stump; regenerate; distal stump) 16 weeks post-implantation. They showed that myelinated fibers (stained green; pointed by green arrows) are regenerating inside the nerve-guide, but fibrosis and debris scavenging are already well advanced in the distal stump. MT staining highlighted the collagen fibers in dark blue. The transverse section of the nylon monofilament suture is identified with an asterisk, and remnants of the suture appeared in pale blue. The area where the wall of the artificial nerve guides were present (ng) showed that the guide was mostly degraded, as a result of the histological processing (ethanol passages). An area of macrophagic infiltration and debris digestion is identified in the distal stump (m). (magnification ×20; scale bar 100 μm)

While the goal of this paper is to provide a detailed protocol of a sciatic nerve gap-injury model in the rabbit, resources employed (animals, consumables, and facilities) benefited from the associated experiments on nerve regeneration in guides longer than 20 mm. In this regard, the main preliminary finding was the documentation of nerve regeneration of myelinated fibers on a distance as long as 26 mm; however, the fibrosis at the distal stump was already overwhelming. How this will prevent, or greatly hinder, the distal progress of regeneration is a matter of further investigation (Fig. 8).

## Discussion

We propose the more translational but still affordable rabbit sciatic nerve model to replace the rat sciatic nerve model for the study of nerve regeneration in gap-injury. While the model has been developed to test artificial nerve guides, it remains a gap-injury model suitable to test other gap-bridging strategies such as allografts or to study the evolution of the gap itself, as in the evolution of a neuroma. Testing our approach in a real applicative example showed that: (1) surgery for testing artificial nerve guides in a rabbit sciatic nerve gap-injury model can be performed in about 30 min, (2) a specific 3D printed tool can assist in the sharp cut of the nerve, (3) rabbits can fully recover in <24 h, (4) about 50% of the rabbits do not exhibit any self-mutilation behavior, (5) the remaining 50% exhibit episodes of self-mutilation of variable severity, of uncertain origin but also mostly treatable, (6) a standardized procedure to sample regions of interest can be used that provides histological slides for an easier comparative reading, and (7) the concurrent immunostaining of MBP and ChAT is possible.

Literature reports state that axonal degeneration and self-mutilation result as a complication of the intramuscular use of ketamine and xylazine in rabbits [55, 56] but this is not our experience. Our monitoring of the self-mutilation episodes seems to suggest that they can occur in a quite serendipitous fashion. They can occur anytime and irrespective of factors like postoperative pain or advancement in the regeneration process. This provides warns to be vigilant and provide a constant two times or three-times daily check of the animal.

Self-mutilation was addressed with a combination of prevention and treatment of the damage with the more severe cases being humanely euthanized. Surgical treatment could be used to address self-mutilation injuries, but a choice between euthanasia and surgical treatment should be based on factors that are related to the overall humane care and use and experimental design. For example, we would not recommend a surgical treatment in an experiment of short duration. The design of scientific end-points which are distributed longitudinally along weeks also provides a way to minimize the loss of animal per end-point. In our case, no animal was lost as it was possible to swap end-points in those cases that required euthanasia.

Our objective was to design a comprehensive protocol to test artificial nerve guides in the rabbit. This protocol will likely improve on previously published reports that indicated, for example, the use of fine scissors to cut the nerve compared to our novel use and demonstration the disposable 3D printed TASC device, or the longer and more complex use of single-arm suture instead of the double-arm suture. While neither the TASC nor the double-arm suture should be considered indispensable, they will certainly contribute to shorten the average duration of the surgery. The plexiform structure and the different elastic properties of nerve components (nerve fibers; perineurium; vessels; epineurium) makes peripheral nerves very resilient to any attempt to cut it sharply by blades or scissors. Accurate surgical technique may succeed in this task; however, we searched for a dedicated surgical instrument able to facilitate and standardize the cutting procedure. The TASC instrument is not only useful to assist the trimming of cut-ends in gap lesions, but it is particularly well suited for the sharp cut of an intact nerve as it is required in the harvesting of a donor autograft, in taking a whole nerve biopsy and in producing experimental gap lesions. We used a double-arm suture, as opposed to a single-arm suture. This allows for easier and faster anchoring of the nerve stump and a faster accommodation of the nerve stumps inside the guide (by a gentle pull of both arms). It also decreases the time that the nerve stump is exposed outside the nerve-guide. The suture transfixed the epineurium prior to the cutting of the nerve, providing a further reduction in the time needed to anchor the stumps, and reducing their manipulation and possible damage. Overall, a reduction in step number and/or in step duration led to an overall shorter surgery time.

In proposing our retrieval procedure, we wanted to establish a consistent method for sampling the nerve in well-defined regions. Our results suggest that just 3 regions of interest (instead of 5) are enough to describe the events in regeneration (as there is an overlap between regions 1 and 2, and between regions 4 and 5 respectively).

A final consideration regards the strict regulations that are enforced by the Unites States Department of Agriculture (USDA) for procedures performed on specific animal species (in our case: the rabbit). The required higher standards of care might impact on the costs and the qualification of the personnel that must be employed. This, however, contributes in our opinion to the greater translation potential of the model, which adopts standards closer to those applied in clinical practice.
